# Intracranial EEG Biomarkers for Seizure Lateralization in Rapidly-Bisynchronous Epilepsy After Laser Corpus Callosotomy

**DOI:** 10.3389/fneur.2021.696492

**Published:** 2021-10-08

**Authors:** Simon Khuvis, Sean T. Hwang, Ashesh D. Mehta

**Affiliations:** ^1^Department of Neurosurgery, Donald and Barbara Zucker School of Medicine at Hofstra/Northwell, Manhasset, NY, United States; ^2^Feinstein Institutes for Medical Research, Northwell Health, Manhasset, NY, United States; ^3^Department of Neurology, Donald and Barbara Zucker School of Medicine at Hofstra/Northwell, Manhasset, NY, United States

**Keywords:** high-frequency oscillation, interictal epileptiform discharge, phase-locked high gamma, epilepsy surgery, corpus callosotomy

## Abstract

**Objective:** It has been asserted that high-frequency analysis of intracranial EEG (iEEG) data may yield information useful in localizing epileptogenic foci.

**Methods:** We tested whether proposed biomarkers could predict lateralization based on iEEG data collected prior to corpus callosotomy (CC) in three patients with bisynchronous epilepsy, whose seizures lateralized definitively post-CC. Lateralization data derived from algorithmically-computed ictal phase-locked high gamma (PLHG), high gamma amplitude (HGA), and low-frequency (filtered) line length (LFLL), as well as interictal high-frequency oscillation (HFO) and interictal epileptiform discharge (IED) rate metrics were compared against ground-truth lateralization from post-CC ictal iEEG.

**Results:** Pre-CC unilateral IEDs were more frequent on the more-pathologic side in all subjects. HFO rate predicted lateralization in one subject, but was sensitive to detection threshold. On pre-CC data, no ictal metric showed better predictive power than any other. All post-corpus callosotomy seizures lateralized to the pathological hemisphere using PLHG, HGA, and LFLL metrics.

**Conclusions:** While quantitative metrics of IED rate and ictal HGA, PHLG, and LFLL all accurately lateralize based on post-CC iEEG, only IED rate consistently did so based on pre-CC data.

**Significance:** Quantitative analysis of IEDs may be useful in lateralizing seizure pathology. More work is needed to develop reliable techniques for high-frequency iEEG analysis.

## Introduction

It is increasingly recognized that a failure of visual inspection to reveal subtle features in intracranial EEG (iEEG) recordings may underlie ambiguity in localizing the epileptogenic zone in surgical epilepsy patients. A number of reports have emerged describing increased confidence in defining the seizure onset zone (SOZ) or epileptogenic zone using quantitative metrics (1–10).

Interictal epileptiform discharges (IEDs) have long been recognized as biomarkers of epileptogenicity (11–14), with the region of brain producing IEDs described as the irritative zone (IZ) (15). The IZ is often larger than the SOZ, and while its complete resection is not necessary to stop seizures, (12) a resection that includes both the SOZ and IZ predicts favorable surgical outcome (16, 17). While the localizing potential of IEDs remains under investigation (5), they appear to have strong lateralizing value in temporal lobe epilepsy (18). Some CC patients in whom there is a consistent (possibly propagation-related) time delay in IEDs occurring between hemispheres ultimately show lateralization of IEDs to the leading hemisphere (19); we explore this in Appendix A. Interictal high frequency oscillations (HFOs) in the 80–500 Hz range often occur at areas of seizure onset independently of low-frequency activity (6, 20). In retrospective studies, resection of regions producing HFOs >250 Hz correlated with better surgical outcomes than zones producing IEDs (4, 21), however, a 2014 Cochrane Review concluded that there was insufficient evidence at this time to use HFOs in surgical planning (22), and several recent studies have challenged the reported special utility of HFOs in defining the epileptogenic zone in individual patients (23, 24).

Schevon and coworkers have reported distinct seizure “core” and “penumbra” regions in mouse models and human patients. The seizure core is characterized by hypersynchronous neuronal firing, which manifests as high gamma (80–150 Hz) electrographic activity coupled to the phase of ongoing low-frequency EEG (25, 26). This opens the possibility that phase-locked high gamma (PLHG) during seizure onset could identify a epileptogenic zone better than human interpretation of low-frequency iEEG. In a recent retrospective analysis of 45 patients, Weiss et al. (27) concluded that resection of early channels showing high PLHG, but not high gamma amplitude (HGA), during seizure onset predicted good surgical outcome at a level non-inferior to that of the manually-labeled SOZ. The extent of channels showing elevated PLHG was more limited than those showing low frequency (2–25 Hz) epileptiform activity, as measured by line length (LFLL), which the authors used as an “objective measure approximating seizure spread as viewed in EEG” (27).

We retrospectively examined the pre-operative iEEG in three subjects with seizures exhibiting very rapid bisynchrony, in whom seizures lateralized after corpus callosotomy (CC) [as in previous reports (28–30)] in order to attempt to identify the hemisphere containing the epileptogenic zone. Patients underwent CC utilizing laser interstitial thermal therapy (LITT) (31) without craniotomy, concurrently with stereoelectroencephalography (sEEG) leads in place bilaterally and symmetrically, firmly affixed to bone [also see report by Silverberg et al. (32)], allowing anatomical correspondence between pre-CC and post-CC measures.

The objective was to assess the utility of quantitative biomarkers in predicting which hemisphere contained the SOZ using the ambiguous pre-CC data, attempting to mimic real-world conditions in which such algorithms may be useful (i.e., cases in which an aide to visual inspection might be desirable). The post-CC data serve as a ground truth and as a positive control. We emphasized individual-level analysis, both because CC cases with iEEG are infrequent and because any useful biomarker should be clinically relevant, with effects that are apparent on the individual-patient level, as has been recognized in recent studies of HFOs (23, 24). Indeed, claims that HFOs (33) and other biomarkers might be useful clinically, on the level of individual patients, motivated our investigation of this small sample set. To our knowledge, the current study is the first to test the effectiveness of intracranial EEG biomarkers on CC patients.

## Materials and Methods

### Clinical Procedure

Informed consent was obtained from three adult subjects undergoing iEEG monitoring for suspected focal epilepsy with rapid bisynchrony and widespread ictal changes (bihemispheric) on scalp EEG at the Northwell Health Comprehensive Epilepsy Center at North Shore University Hospital. Focal tonic and focal bilateral to tonic-clonic seizures were suspected because each subject presented with either a structural lesion or semiological features suspicious for focal onset. Even in cases of structural lesion, concerns remained due to bihemispheric EEG data that the lesion may not have corresponded strictly with the epileptogenic zone, and lesionectomy risked missing surrounding epileptogenic tissue or potential multifocality associated with kindling. Furthermore, Subject 3 had bilateral lesions, Subject 2 had a history of transcallosal surgical resection of a midline lesion with bilateral interhemispheric retraction, and Subject 1 had no structural lesion according to which to define surgical margins. For all of these reasons, the risk-benefit ratio of iEEG was deemed favorable by the clinical team. Subject characteristics can be seen in Table 1.

**Table 1:** Table of enrolled subjects.

**Subject#**	**1**	**2**	**3**
Age	28	23	44
Sex	F	F	M
Age of onset	5 months	17 years	29 years
Handedness	R	R	R
Type of epilepsy	Suspected focal	Suspected focal	Suspected focal
Etiology of epilepsy	Unknown etiology	Right frontal encephalomalacia (secondary to interhemispheric approach to craniopharyngioma)	Left frontal encephalomalacia with hemosiderin deposition. Right temporal cavernoma; pheochromocytoma
Type and frequency of Szs	Focal aware, tonic, FBTCS	Tonic, FBTCS	Focal impaired awareness, FBTCS
Semiology	Tonic bilateral stiffening, late head turn to left	Tonic bilateral stiffening, dystonic posturing of arms	Bilateral convulsions, late right head version, aphasia
Medications	Lamotrigine 150 mg bid, Oxcarbazepine 600 mg bid	Levetiracetam 2,000 mg bid, Zonisamide 100 mg bid	Carbamazepine XR 600 mg bid, Clobazam 30 mg Bedtime, alprazolam 6 mg per day
Structural MRI	Normal	Right frontal encephalomalacia, midline sellar mass	3-cm Left frontal hemosiderin deposition and encephalomalacia; 0.5-cm right anterior temporal cavernous malformation
Type of electrodes	sEEG	sEEG	sEEG
Lateralization of Ictal EEG, pre-CC	Bilateral onset, non-lateralized frontal × 2	Bilateral paracentral onset, right evolution	1 Left and 2 non-lateralized bifrontal
Lateralization of Ictal EEG, post-CC	Right frontal × 4	Right-sided evolution, frontoparietal	Left frontal × 2
Lateralization of Interictal EEG, pre-CC	Bilateral multifocal: left frontal and right frontal, frequently synchronous, right medial temporal	Bilateral multifocal: bisynchronous frontal, right frontal, left frontal, left medial temporal	Bilateral multifocal: left frontal, left medial temporal; left lateral temporal, right frontal, right medial temporal
Lateralization of Interictal EEG, post-CC	Bilateral multifocal: right frontal >> left frontal, right medial temporal	Bilateral: right frontal > left frontal	Bilateral multifocal
Outcome (Engel I-IV)	IIA (R frontal lobectomy) Three seizures in 5 years, one while off medications	III (R frontal lobectomy/lesionectomy). Initially seizure-free for 1 year, then experienced tumor recurrence with new seizure activity and diabetes insipidus by 5 years.	IC Three seizures in last 5 years, last occurring 2 years ago
Total number of analyzable channels	92L/91R	109L/119R	113L/124R

*FBTCS, focal to bilateral tonic clonic seizure; sEEG, stereo EEG; CC, corpus callosotomy; h/o, history of; mo, month; Sz, seizure; R, right; L, left; bid, twice daily*.

EEG macro depth electrodes (PMT Corporation, Chanhassen, MN) were implanted bilaterally and symmetrically, and fixed to the skull with anchor bolts, in accordance with clinical protocol. Localizations of electrodes were produced with the aid of freely-available software (34–40), and can be found in Appendix B. Electrodes were named with a letter code starting either with “L” for left or “R” for right. The subsequent letters correspond to the electrode's intended trajectory within its respective hemisphere, and occur in matching pairs as a result of the symmetrical implants. In Subject 1, 11 depth electrodes were implanted on each side, with a total of 189 usable channels. Six electrodes targeted frontal cortex on the left (covering medial and lateral areas), three electrodes sampled parietal cortex, one (LDh) sampled amygdala and also insula and lateral temporal cortex, another (LFI) sampled insula along with lateral frontal cortex. On the right, the distribution was the same, except that RDh was closer to the hippocampus than the amygdala. In Subject 2, nine depth electrodes were implanted on each side, with a total of 224 usable channels. On the left, five depth electrodes sampled frontal cortex, two sampled parietal cortex, one (LDa) sampled the hippocampus and lateral temporal areas and one (LFI) sampled the insula, by way of the frontal cortex. On the right, the distribution was the same, except that RFp sampled both the frontal and parietal cortices, while LFp sampled only the parietal cortex. Subject 3 was implanted with ten depth electrodes on each side with a total of 224 useable channels. On the left, five depth electrodes sampled medial and lateral frontal cortex, one (LP) sampled parietal cortex, LDh, and LDa sampled hippocampus and amygdala *via* lateral temporal cortex, respectively, one (LTx) sampled the lateral temporal cortex and one (LI) sampled both the frontal and temporal cortices. On the right, RFp spanned pre-, post-, and paracentral gyri (whereas LFp mainly sampled the precentral), and (in addition to temporal and frontal cortices) RI also sampled the insula.

EEG data were recorded and monitored continuously using an XLTek system (Natus Medical Corp., Pleasanton, CA), at 512 Hz sampling rate; the system has an anti-aliasing filter with a 200 Hz 3-dB cutoff. Antiepileptic drugs were held, and at least two seizures were recorded from each subject. Seizure and interictal data were reviewed by a board certified epileptologist. Subjects were recommended for CC solely on clinical grounds. All subjects underwent MRI-guided LITT ablation of the anterior two thirds of the corpus callosum using the Visualase system (Medtronic, Minneapolis, MN) with electrodes in place. Electrodes were firmly fixed to the skull and did not move during the procedure. The MRI compatibility of depth electrodes under the conditions used has been verified independently with phantom models (41), and subjects showed no evidence of thermal injury clinically or in subsequent imaging. Subjects were monitored post-CC until at least one additional seizure was recorded from each. Follow-up outcomes were assessed at 5 years. Seizure onset and interictal EEG data were analyzed retrospectively using custom code for MATLAB (MathWorks, Natick, MA). All procedures were approved by the Institutional Review Board of the Feinstein Institutes for Medical Research, Northwell Health.

### Interictal Data

Four pre-operative and two post-operative interictal intervals, of 30 min each, balanced equally between “Wake” and “Sleep” periods, were chosen for each of the three subjects. Wake periods were selected by reviewing video footage of each subject's monitoring stay and selecting intervals during which the eyes were open continuously, while minimizing the time that the subject was moving, speaking or interacting physically with visitors or staff. Lacking scalp EEG data, and therefore an electrophysiologic confirmation of sleep, additional intervals were identified during which the each subject was thought to be asleep based on eye closure and lack of movement, interactivity or signs of wakefulness on video recorded simultaneously with EEG. We designated these intervals as “Sleep” periods, acknowledging the inherent limitations in choosing data without the aid of scalp EEG confirmation. All chosen intervals were at least 6 h from any seizure, and a minimum of 6 h after any direct electrical brain stimulation procedure. All recordings were visually reviewed for good signal quality. Channels with high line noise or electrical artifact were removed after visual inspection, and all remaining channels were referenced to either a common (Subject 3) or local electrode (Subjects 1 and 2) average, depending on the severity of the noise. Channels used for each subject (along with channels localized to the structural lesions of Subjects 2 and 3) are tabulated in Appendix C.

Subject 1 was on lamotrigine 150 mg, twice daily, and oxcarbazepine 600 mg, twice daily, tapered down to lamotrigine 50 mg, twice daily. After two seizures, home medications were restarted in anticipation of CC, and pre-CC interictal samples were recorded during this medicated period. After CC, medications were again tapered to elicit more seizures, and post-CC interictal intervals were recorded on 50 mg of lamotrigine and 300 mg of oxcarbaizpine, twice daily.

Subject 2 was on levetiracetam 2,000 mg, twice daily, and zonisamide 100 mg, twice daily. The subject seized spontaneously with levetiracetam 2,000 mg, twice daily, and a total daily dose of zonisamide of 100–300 mg, both pre- and post-CC. All interictal intervals were obtained on this medication range.

Subject 3 was tapered off of oxcarbazepine, clobazam and alprazolam pre-operatively, with pre-CC interictal intervals taken when the subject was close to the end of the taper (Pre-CC Sleep Baseline 1) or on 0.25–0.5 mg of alprazolam daily and no anti-epileptic agents (all other baselines). Subject 3 was restarted on carbamazepine 600 mg, twice daily, prior to and immediately following CC, then again tapered to elicit more seizures. Post-CC Wake Baseline 1 was recorded when the subject was on carbamazepine 200, twice daily, and alprazolam 0.5 mg, twice daily; Post-CC Sleep Baseline 1 was recorded when the subject was only taking alprazolam 0.25 mg, twice daily.

### High-Frequency Oscillations

**Figure 1:**
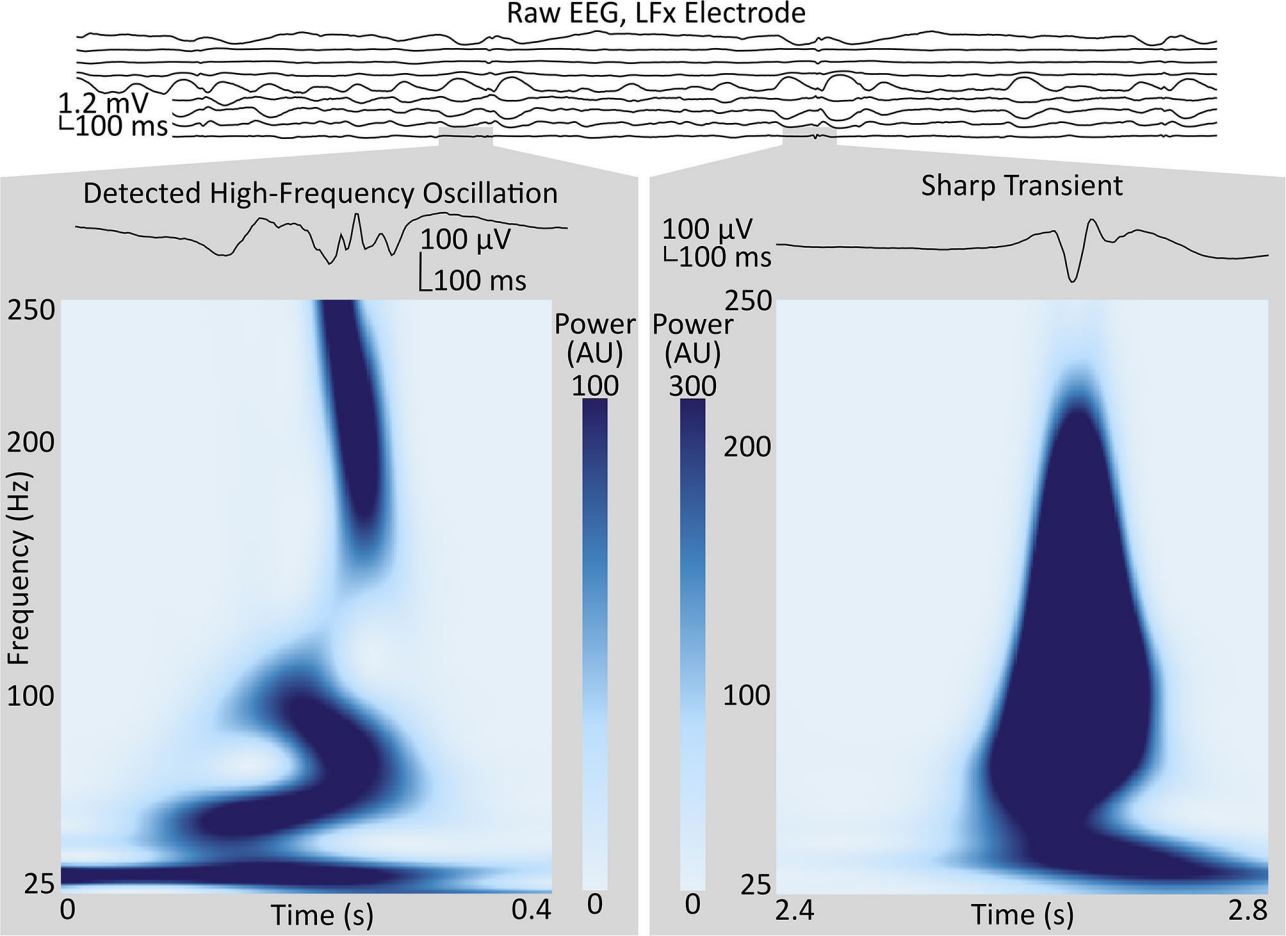
Sample of intracranial EEG from the LFx depth electrode implanted in Subject 1, targeting the left frontal pole, with bipolar-referenced signals from contact pairs shown in spatial order, with the most-terminal contact on top. Two segments from the LFx9–10 (bottom) channel, in the anterior middle frontal gyrus, illustrate high-frequency events positively identified as an HFO (left), and not identified as an HFO (right), respectively, by the technique described in *Methods* (section High-Frequency Oscillations). Gray boxes show the enlarged waveforms and the spectrograms, as calculated by the Stockwell transform, of each segment. Contrast the multiple oscillations in the voltage trace and the trough in the power spectrum around 40 Hz in the segment identified as an HFO to the single high-amplitude voltage spike and the broadband power increase in the segment rejected by the algorithm. HFO, high-frequency oscillation.

The algorithm of Brunos et al. (42) was adapted to detect HFOs. Briefly, the algorithm consists of two stages: first, defining “events of interest,” and, then, identifying HFOs among these events. The events of interest were identified by filtering the signals from each channel between 80 and 500 Hz with an infinite-impulse-response Cauer filter with 60 dB attenuation at the corner frequencies, 0.5-dB pass band ripple and 10-Hz upper and lower transition widths, in both forward and reverse directions, then specifying a threshold three standard deviations above the mean analytic signal amplitude (*via* Hilbert Transform) of the so-obtained bandpass-filtered signal, over a duration of 5 min. Though 3 is the default proposed by Brunos et al., values for the threshold between 3 and 10 were evaluated in our paper. Times when the filtered signal envelope exceeded the threshold and the duration between half-threshold crossings was at least 6 ms, qualified as events of interest. Events of interest with inter-event intervals of <10 ms (changed to 100 ms in our code) were merged, and events wherein the (rectified) bandpass-filtered signal did not have at least six peaks above 2 standard deviations above the mean were rejected. The Stockwell transform of the signal contained within the full-width-at-half-maximum of the envelope was inspected for two separate islands of power: one with a peak at or above 60 Hz, a trough (minimum value) below that peak and above 40 Hz, and another peak at the first local maximum below that trough. To be classified as an HFO, the tough has to be deep enough (20% of the dB-transformed power of the high-frequency peak, in keeping with the procedure for Brunos et al.) and the high-frequency peak must be at least twice as powerful as the low-frequency peak (relative to the trough), for the entire full-width-at-half maximum period (Figure 1).

Because HFOs are highly focal phenomena (10, 43), we rejected all events that co-occurred within a single electrode array (ranging from 4 to 8 cm in length and including 8–16 contacts). We used a robust half-maximum, by halving the mean of the HFO counts of the five channels with the highest HFO rates, as a threshold. A generalized linear model was employed, using the number of channels above the threshold (relative to the total number of channels) in each hemisphere during each baseline recording as response variables and the laterality (more- or less-pathologic) and recording number as categorical explanatory variables. A binomial distribution for the explanatory variables was assumed (given that they represent ratios of channels) and a logit link function was used. The MATLAB function fitglm() as used to assess the significance of the effect of laterality. Statistical significance was declared at *p* < 0.05, with Bonferroni correction at *N* = 3, for three subjects.

### Interictal Discharges

The algorithm of Janca et al. (44) was used for detecting IEDs. Briefly, each channel is filtered between 10 and 60 Hz using a combination of high- and low-pass 8th order zero-phase type II Chebyshev filters. Line noise is removed with a notch biquad filter at 60 Hz with a 4-Hz stop band. The analytic signal (*via* Hilbert Transform) envelope is analyzed in 5-s moving windows with 80% overlap, and a log-normal distribution is fit to the amplitude data from each segment using a maximum-likelihood estimator (MLE). A threshold is calculated from the sum of the median and mode of the distribution, scaled by an empirically-determined coefficient. The thresholds for all of the segments are interpolated using a cubic spline and smoothed with a moving-average filter. Spikes are defined as threshold crossings, with multiple crossings within 120 ms combined into a single event. Beta and mu oscillations trigger false detections, so intervals with spectral peaks between 10 and 25 Hz are identified and omitted.

IEDs were labeled unilateral if their extent was limited to a single hemisphere. A maximum likelihood estimator (MLE) on the binomial parameter *p*, representing the relative frequency of unilateral IEDs on the more pathologic-hemisphere, was calculated for each subject. Statistical significance was declared at *p* < 0.05, with Bonferroni correction at *N* = 3, and relative to the stricter of two chance criteria: the relative fraction of electrodes on the more-pathologic side, and 50%.

The procedure was repeated after removing all channels corresponding to electrode contacts located within the structural lesions in Subjects 2 and 3, and all electrode contacts contralateral to those removed at this stage or in the above analysis because of high noise or extra-parenchymal location, leaving data recorded from symmetrical sites.

### High Gamma and Phase-Locked High Gamma

EEG from seizure onsets were filtered to remove 60 Hz and harmonics with 8th-order zero-phase IIR notch filters. Data were either referenced to a common average (Subject 3) or a local (electrode) average (Subjects 1 and 2), depending on the severity of the noise.

**Figure 2:**
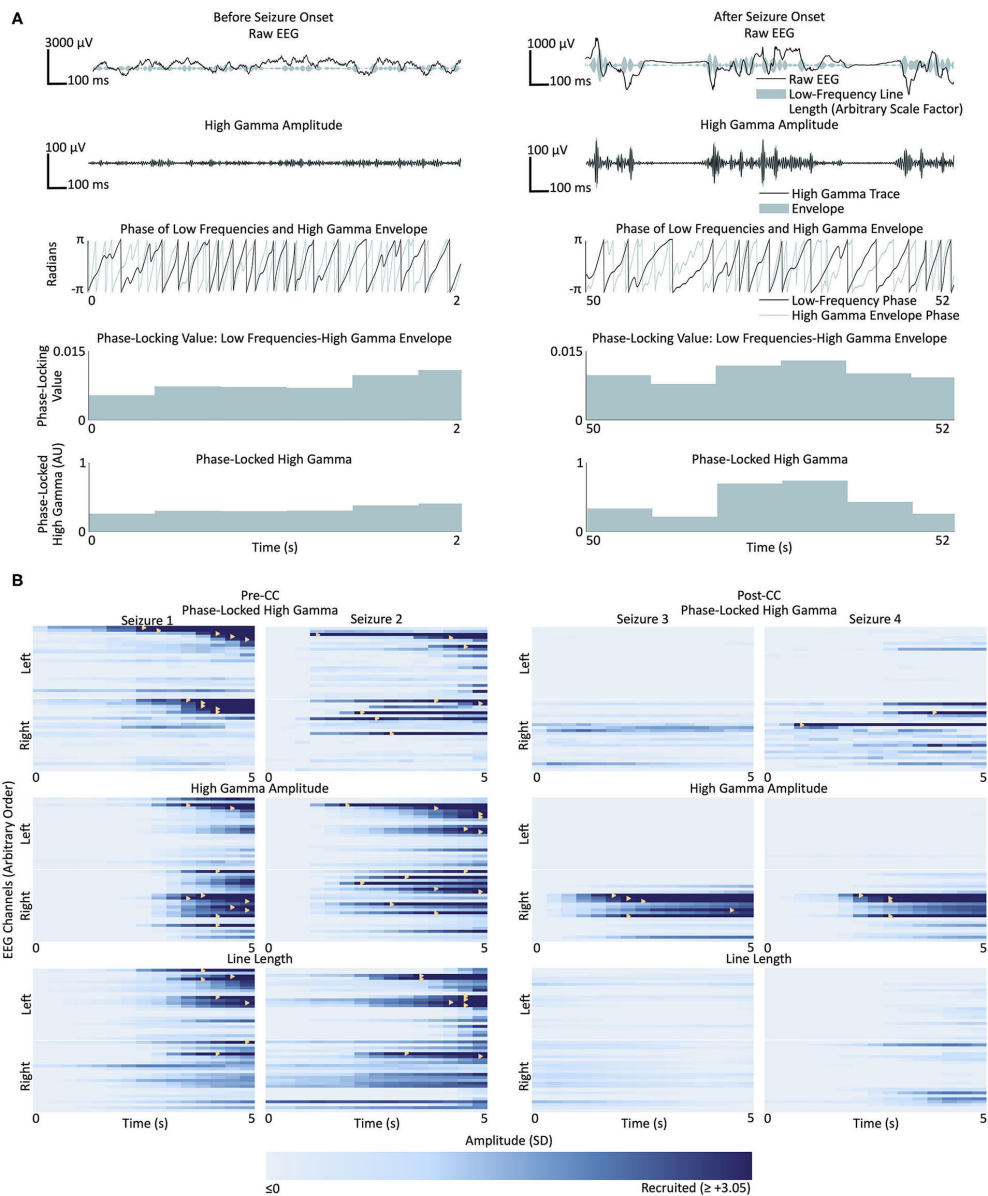
**(A)** Representative phase-locked high gamma calculation from two sections of EEG from a single channel in Subject 1, before (left) and after (right) recruitment into Seizure 1. Top row: raw EEG in black, and low-frequency line length represented by breadth of teal shaded area; second row from top: signal filtered for high gamma (500th-order finite impulse response, 80–150 Hz) in black, and envelope of the high gamma signal in teal; third row: instantaneous phase of the low frequency band (500th-order finite impulse response filter, 4–30 Hz) in black, and high gamma envelope in teal; fourth row from top: phase-locking value between the low frequency signal and high gamma envelope; bottom row: phase-locked high gamma. **(B)** Seizures from Subject 1. Each box represents the time course of seizure evolution, showing phase-locked high gamma (top row), high gamma amplitude (second row) and low-frequency line length (bottom row), The left two columns represent the two seizures before corpus callosotomy (CC), and the right two columns represent the two post-CC seizures. Within each box, each horizontal bar represents the time evolution of a single channel, with channels in the left hemisphere shown above the white line and channels in the right below. Light shades of blue represent pre-recruitment levels of phase-locked high gamma, high gamma amplitude or low-frequency line length, at each time and channel, respectively, with progressively darker shades representing increasing levels up to the threshold of 3.05 standard deviations above the pre-recruitment mean. Time points at which individual channels first surpass the threshold value are denoted by yellow triangles. Line length shows a trend toward left-sided onset pre-CC, however the seizures lateralize to the right post-CC. High gamma measures trend toward right-sided onset pre-CC, especially high-gamma amplitude, and also reach the recruitment threshold earlier in the course of the seizure post-CC than low-frequency line length does. Pre-CC, pre-corpus callosotomy; Post-CC, post-corpus callosotomy.

The methods used by Weiss et al. (27) were followed closely, with limited exception; the protocol is summarized in Figure 2A. The high gamma and low-frequency components of the signal were extracted by applying 500th-order finite impulse response (FIR) bandpass filters between 80 and 150 Hz and between 4 and 30 Hz, respectively. Edakawa et al., report an optimal PLHG low-frequency band range of between 8 and 13 Hz for seizure detection (45), and the algorithm was tested with these values, separately. The phase-locking value is defined as:


$$\begin{array}{l} PLV=\;\frac{1}{N}\overset{N}{\underset{n=1}{\sum }}e^{i\left(\phi_{LF}\left[n\right]-\phi_{HFA}\left[n\right]\right)}, \end{array}$$

where ϕ_*LF*_ is the phase of the analytic signal of the low-frequency component (filtered between 4 and 30 Hz in Weiss et al.'s methods) and ϕ_*HFA*_ is the phase of the analytic signal of the amplitude of the analytic signal of the high-frequency component (filtered between 80 and 150 Hz). We deviated from the protocol of Weiss et al., in applying a zero-phase filter with passband between 4 and 30 Hz to the amplitude of the high gamma analytic signal. This step is recommended to remove DC offsets from the data (46, 47), so that phase information after subsequently-applied Hilbert transforms is meaningful (48). PLHG was derived by multiplying the phase-locking value by the instantaneous HGA. The PLHG in each channel was corrected by the root-mean-square (RMS) HGA in two or four 30-min baseline periods, depending on whether that given subject was awake during their seizures (2 asleep; 2 awake and 2 asleep; and 2 awake for Subjects 1, 2, and 3, respectively). HGA was also corrected by dividing by its baseline RMS value. LFLL, a proxy used by Weiss et al., for human-readable EEG changes at low frequencies (27), was also calculated by taking the absolute value of the first-order difference of the signal obtained by filtering the EEG between 2 and 25 Hz with a 500th-order FIR filter and dividing by the baseline RMS value. Again, following the protocol of Weiss et al., the values of each of the three metrics were calculated for 0.3-s intervals in each channel and smoothed over 20 consecutive intervals with a uniform boxcar.

**Figure 3:**
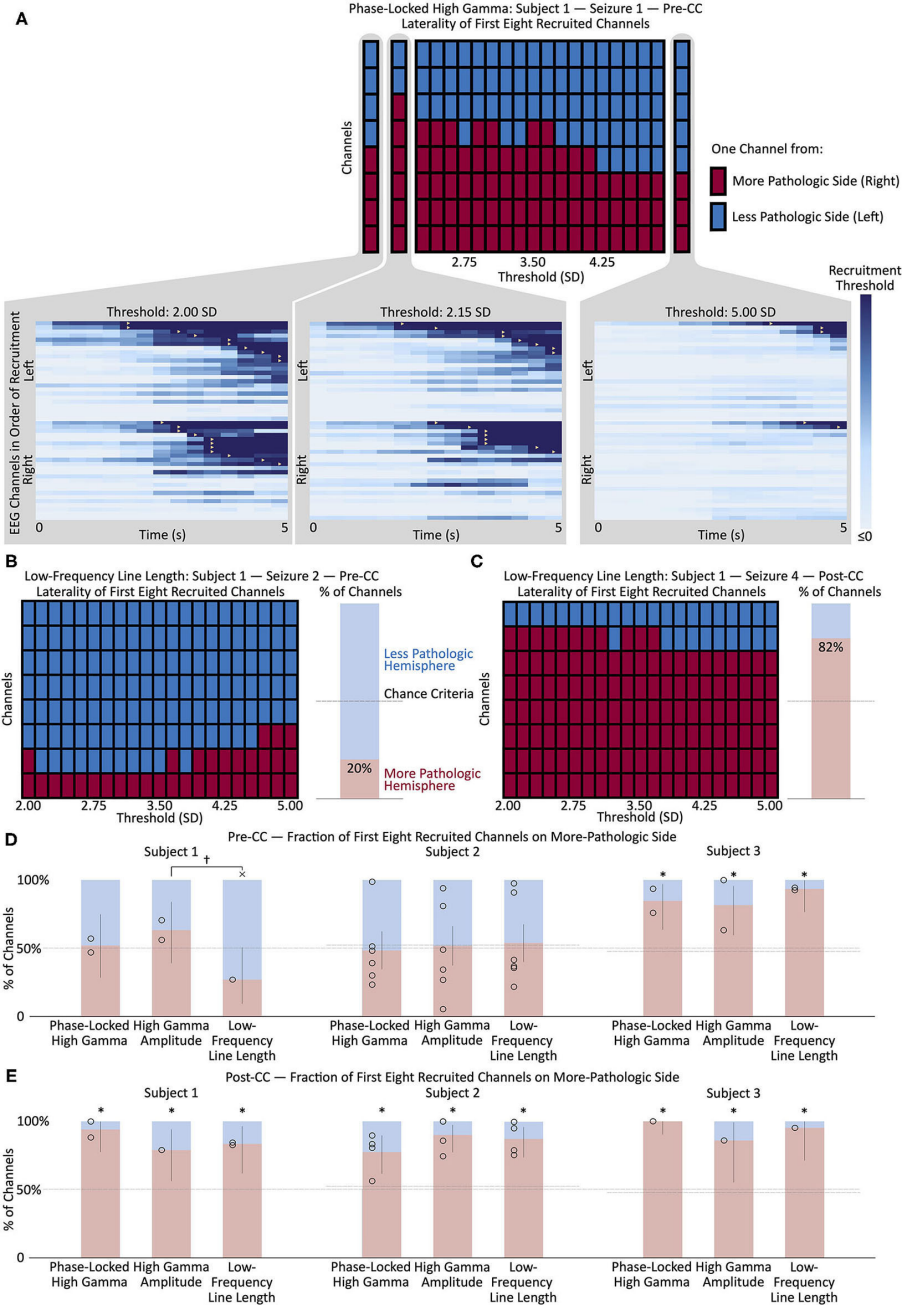
**(A)** Change in the left/right distribution of the first eight channels recruited to a representative seizure (Subject 1, seizure 1) as a function of the recruitment threshold. Each red or blue square represents one channel from the right or left (more- or less-pathologic) hemisphere, respectively, and the horizontal axis shows how the distribution of the first eight recruited channels changes as a function of the recruitment threshold of the phase-locked high gamma value. Insets show the seizure onset, with each horizontal line representing the time evolution of the phase-locked high gamma in one channel, and color scaled to represent 0 through 2.00, 2.15, and 5.00 standard deviations above mean pre-seizure levels, respectively. Recruitment events, where the phase-locked high gamma exceeds threshold, are represented by yellow triangles. Note the change in recruitment patterns as the threshold is modulated. **(B)** Change in the left/right distribution of the first eight channels recruited to a representative pre-CC seizure (Subject 1, seizure 2) as a function of the recruitment threshold, using the low-frequency line length metric. The bar graph on the right shows the mean percentage of the first eight recruited channels in the more-pathologic hemisphere in red, and in the less-pathologic hemisphere in blue, from the domain of thresholds between 2.00 and 5.00. The horizontal dashed line represents the chance conditions of 50% and the percentage of electrodes on the more-pathologic side (almost the same in this subject). Here, the portion of channels on the more-pathologic (correct) side is considerably less than that on the less-pathologic side—low-frequency line length does not correctly lateralize this pre-CC seizure. **(C)** Same as **(B)** but for post-CC seizure 4. Here, there is a strong suggestion of lateralization to the correct hemisphere, as seen in the preponderance of channels from the more-pathologic hemisphere (red squares) among the first eight channels recruited across the domain of thresholds. **(D)** Maximum likelihood estimates and confidence intervals (corresponding to *p* < 0.05) of the fraction of the first eight recruited channels from the more- and less-pathologic hemispheres in red and blue, respectively, across pre-CC seizures using phase-locked high gamma, high gamma amplitude and low-frequency line length metrics in each of the three subjects. Fifty-percent and channels-on-correct-side chance levels shown as dotted lines. **(E)** Same as **(D)** but for post-CC seizures. *p < 0.05, Bonferroni corrected for *N* = 3 subjects, relative to the stricter of the two chance performance levels. ^†^*p* < 0.05, uncorrected × wrong side, *p* < 0.05, uncorrected, vs. at least one chance criterion. Pre-CC, pre-corpus callosotomy; Post-CC, post-corpus callosotomy.

Channels were considered “recruited” into the seizure when the amplitude of the given measure exceeded a variable threshold. The recruitment pattern was repeatedly calculated, using each of 21 evenly-spaced threshold values between 2.0 and 5.0. Figure 2B illustrates the recruitment of channels into four seizures from Subject 1 according to PLHG, HGA, and LFLL metrics. This was done to avoid choosing an arbitrary threshold value, since recruitment patterns can change considerably with different thresholds (see Figure 3A). An MLE of the fraction of channels on the more-pathologic side was calculated for each metric for each subject (example shown in Figure 3B), and compared at α = 0.05, Bonferroni corrected *N* = 3 with the stricter of the fraction of channels on that side, or 50% chance level, as shown in Figures 3B,C. The performance of the measures was also compared to each other at α = 0.05.

## Results

### Patient Outcomes

**Figure 4:**
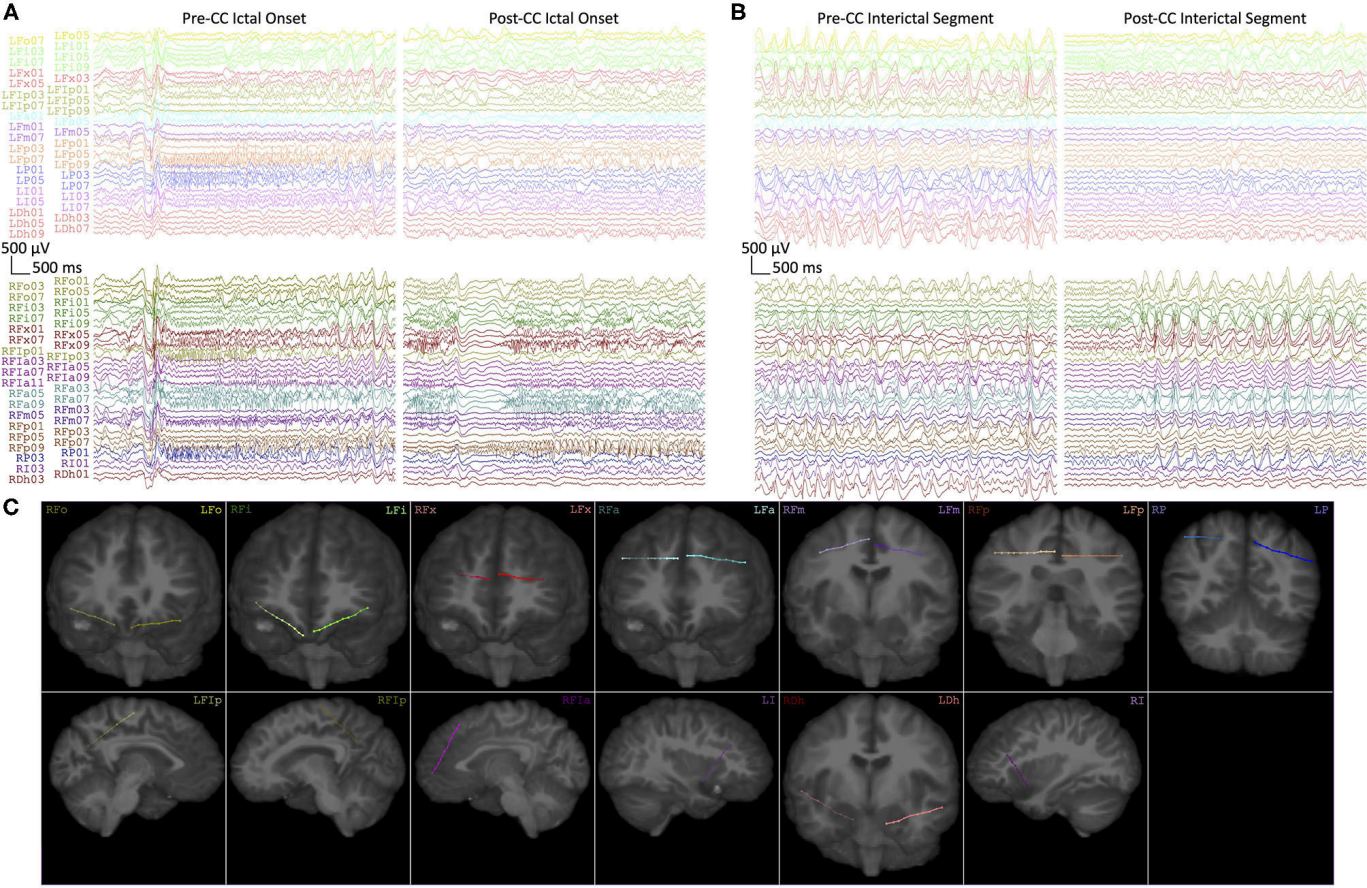
**(A)** Representative intracranial EEG of seizure onsets from Subject 1 before (Pre-CC) and after (Post-CC) corpus callosotomy (seizures 1 and 6, respectively). Each electrode array is represented by a different color, with left-sided arrays on top and right-sided arrays on the bottom. The pre-CC seizure shows bilaterally-synchronous onset, whereas the post-CC seizure exhibits onset and evolution on the right. **(B)** Representative segments of interictal EEG from Subject 1 before (Pre-CC) and after (Post-CC) corpus callosotomy, channels represented as in **(A)**. The pre-CC EEG exhibits widely-distributed bilateral pathology, whereas the post-CC EEG shows lateralization of interictal pathology to the right. All segments referenced to a common average and filtered between 1 and 59 Hz with a 8th-order zero-phase Butterworth filter. **(C)** Reconstructions of the implantation locations of all of the electrode arrays in **(A,B)**, with deeper channels represented by lower numbers, the terminal contact being numbered “1.” Contacts outside of the plane of the image are represented by proportionally-decreased alpha opacity.

Patient outcomes for all three subjects are described in Table 1. All three subjects showed lateralization of their seizure onsets after CC, and Subject 1 also showed a very strong subsequent lateralization of IEDs, as seen in Figure 4. Subjects 1 and 2 went on to have resections of the suspected epileptogenic cortex, while Subject 3 had substantial improvement in seizure severity with CC alone, and declined further surgical treatment that would have required open craniotomy.

### High-Frequency Oscillations

**Figure 5:**
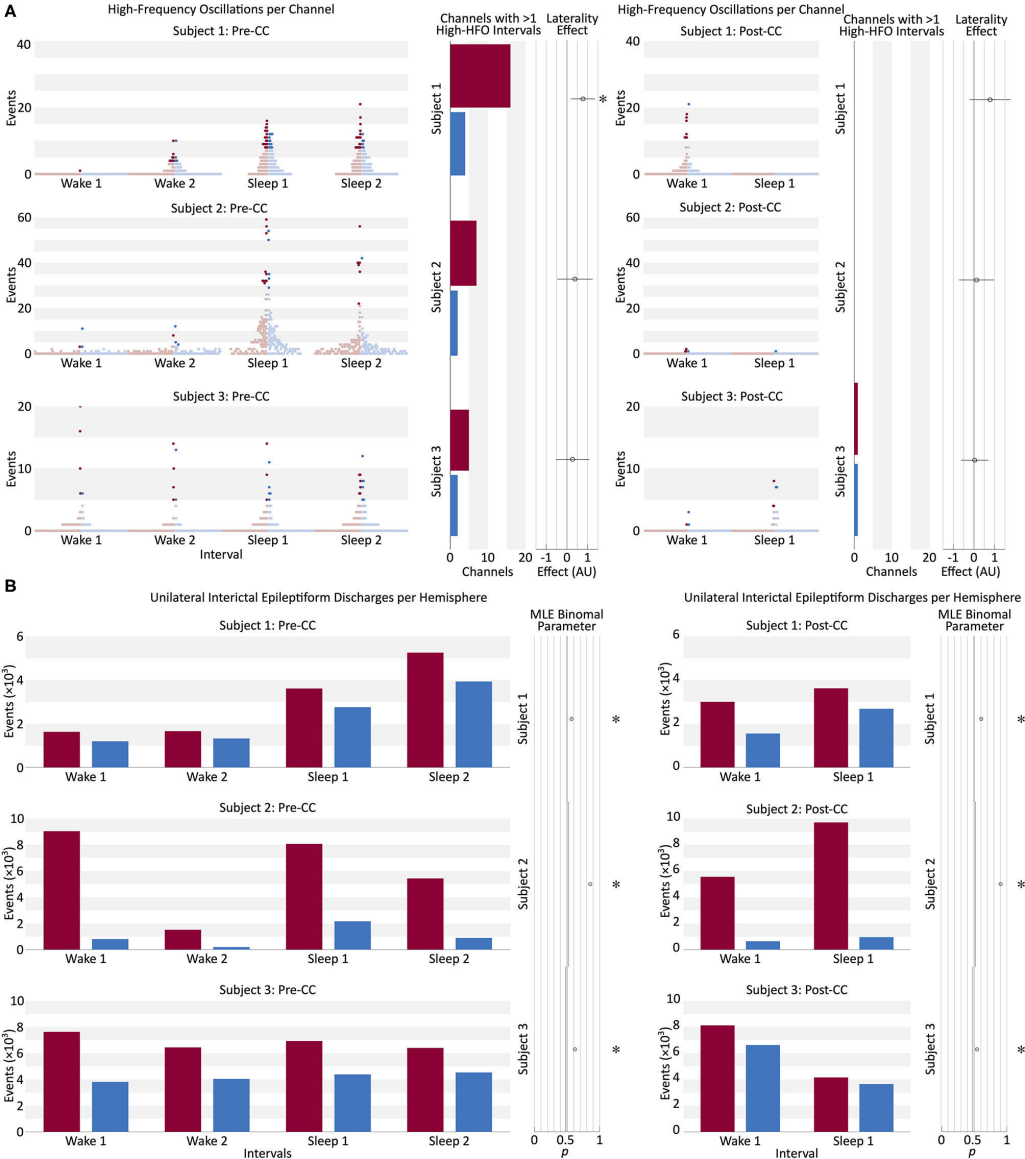
**(A)** Beeswarm boxplot representing the distribution of HFO counts per channel in each of four pre-CC and two post-CC 30-min baseline segments of interictal intracranial EEG in the three subjects. Two pre-CC and one post-CC baselines from each subject were taken from periods of quiet wakefulness (Wake), and the same number from periods of sleep (Sleep). Each red dot represents a single EEG channel from the hemisphere identified post-CC as predominantly pathologic, and each blue dot represents a channel from the contralateral hemisphere. The intensely-colored dots represent channels that meet the robust half-maximum criterion, and the pale dots represent those that do not. The bar graphs for each subject show the number of channels from the more-pathologic (red) and less-pathologic (blue) hemispheres that met the robust half-maximum criterion during at least two of the baseline intervals. The circle markers show the effect of laterality in a general linear model fit to the fraction of channels exceeding the robust half-maximum criterion on each side, with positive values representing a greater fraction of high-HFO channels in the more-pathologic hemisphere. Error bars: 95% confidence intervals, *: *p*_Type I Error_ < 0.05, Bonferroni corrected for *N* = 3 subjects. HFO, high-frequency oscillation; Pre-CC, pre-corpus callosotomy; Post-CC, post-corpus callosotomy. **(B)** Number of unilateral interictal epileptiform discharges from the more- and less-pathologic hemispheres over the same 30-min intervals as in part A, in red and blue, respectively. Circle markers show the maximum-likelihood fit value of binomial parameter *p* to the fraction of discharges on the more-pathologic side. Error bars: 95% confidence intervals, *p_Type I Error_ < 0.05, Bonferroni corrected for *N* = 3 subjects. Comparison to stricter of two chance performance levels: hemispheres and channels on more pathologic side (see *Methods* section Interictal Discharges). Pre-CC, pre-corpus callosotomy; Post-CC, post-corpus callosotomy.

Interictal data showed large variation in HFO rates among pre-CC intervals within individual subjects (Figure 5A). The more-pathologic hemispheres had a trend toward a greater fraction of high-HFO channels in all subjects. Fitting a general linear model showed a significant effect of pathology laterality on the fraction of high-HFO channels in 1/3 subjects in the pre-CC intervals. Post-CC intervals also showed a trend toward a laterality effect linking the fraction of high-HFO channels to the more-pathologic hemisphere in all subjects, but this trend did not reach significance. Changing the threshold for HFO detection from ten to three considerably increased the number of HFOs, and resulted in significant lateralization of Subject 1's post-CC HFOs to the correct side, however, it also resulted in a significant lateralization of Subject 2's HFOs to the incorrect side pre-CC (*p* < 0.05, Bonferroni correction at *N* = 3).

Examining only channels above the HFO-rate half-maximum during at least two pre-operative interictal intervals: the fraction of channels on the more-pathologic side trended far above the chance value, however, due to small absolute numbers of channels meeting this criterion in each subject (<20 in each hemisphere), this trend did not survive correction for multiple comparisons (*p* > 0.05). Post-operatively, Subjects 1 and 3 did not have any channels that exceeded the half-maximum threshold during both intervals, and Subject 2 showed no strong trend. High-HFO channels were not more likely to be located within the structural lesion (2/9 high-HFO channels in 44/224 lesion channels in Subject 2, *p* = 0.9, chi-squared test; and 0/8 high-HFO channels in 12/244 lesion channels in Subject 3, *p* = 0.5, chi-squared test). Appendix C tabulates the high-HFO channels in Figure 3.

### Interictal Discharge Counting

Unilateral IEDs were more frequent in the more-pathologic hemisphere in all subjects during all intervals, pre- and post-CC (*p* < 0.05, Bonferroni correction at *N* = 3). CC resulted in greater lateralization in 2/3 subjects, and reduced lateralization in Subject 3 (*p* < 0.05). Pre-CC values of the binomial *p* ranged from 0.58 to 0.82 in favor of the more-pathologic hemisphere (Figure 5B). In Subjects 2 and 3, we investigated whether the effect was attributable to data recorded from within those patients' structural lesions, which would have limited the applicability of this finding to the broader population. The analysis was repeated with channels recoding from intralesional sites excluded, as well as any channel contralateral to an excluded channel. The result were robustly replicated in both Subjects 2 and 3 (Appendix D).

### High Gamma and Phase-Locked High Gamma

Figure 3E shows the post-CC ictal lateralization of the three subjects, using PLHG, HGA and LFLL. As expected, all three metrics showed the correct lateralization in all three subjects. In fact, no individual seizure lateralized to the less-pathologic hemisphere using any of the metrics. No metric lateralized the seizure onset significantly better than any other.

Figure 3D shows the pre-CC ictal lateralization of the three subjects, using PLHG, HGA, and LFLL. Only Subject 3 shows significant lateralization of seizure onsets to the more-pathologic hemisphere using any of the metrics, with all metrics performing above chance and with no significant differences among the three. Subject 1 showed near-significant lateralization of the seizure onsets to the *less-pathologic* hemisphere, with the LFLL metric (*p* < 0.05 relative to the less stringent chance criterion of 50%, without correction for multiple comparisons). There was a trend toward improved performance (*p* < 0.05, not corrected for multiple comparisons) of HGA relative to LFLL, but both HGA and PLHG perform statistically no better than chance. None of the three metrics performed significantly differently from chance, or from one another in Subject 2.

Results did not change radically when the first four and 12 channels were used to calculate the MLE. A weak qualitative trend toward better and worse performance could be seen using the beta EEG range (13–25 Hz) and omitting the filtering step before applying the second Hilbert transform to the high gamma envelope, as in Weiss et al.'s protocol (27), however, no statistically-significant differences were seen, and deviations from the PLHG MLEs calculated by the primary method were small in magnitude. We could not replicate Weiss et al.'s finding that the channels recruited in the first 30 s significantly lateralized the epileptogenic zone. PLHG and HGA both failed to reach significance at α = 0.05, Bonferroni corrected to *N* = 3, and LFLL significantly lateralized to the more pathologic hemisphere only in Subject 2 (data not shown).

## Discussion

Even though CC is most indicated for generalized epilepsy with atonic seizures, its utility for revealing previously-obscured foci in focal epilepsy has been reported (29, 49, 50). We describe a unique series of three subjects whose pre-CC scalp EEG and sEEGs were difficult to lateralize, and whose post-CC seizure onsets became localizable. Specifically, Subject 1 had bilateral seizure onset and interictal activity prior to CC, and seizures lateralized clearly post-CC (Figure 4). Subjects 2 and 3 showed more varying degrees of ictal rapid bilateral synchrony pre-CC.

We used this unique opportunity to evaluate several quantitative iEEG biomarkers on the pre-CC data to see if any of them could have predicted the ultimate lateralization of the patients' seizures, with the ultimate goal of extending our findings to non-CC patients.

We analyzed the three subjects individually because of the small size and the diverse nature of our population. We referred to the subjects' hemispheres as more- and less-pathologic, since we cannot definitively disprove pathology on the contralateral side. While Subjects 2 and 3 showed clear MRI pathology lateralized to the more pathologic hemisphere, there were convincing electrographic signs of bilateral abnormality. However, in all subjects, the difference in the degree of epileptogenicity in the hemispheres was sufficient to implicate a single hemisphere in seizure onset in the post-CC iEEG. Consistent with this idea, subjects 1 and 2 were initially seizure-free for over 1 year after unilateral resection, and subject 3 had only focal impaired awareness seizures after the procedure with seizure onsets from the more pathologic hemisphere.

### Interictal Data

IED and HFO frequencies change with level of arousal, occurring most frequently in non-REM sleep (6, 14), so we sampled both wakefulness and (by observation) sleep.

Electrical noise poses a considerable challenge to HFO identification, so we took steps to mitigate false detections. HFOs that co-occurred throughout the same electrode array were rejected. Given the 4–8 cm length of the array, traversing both gray and white matter, we reasoned that any discharge captured across its length would have been more likely to reflect artifact than HFO. We also did not use the raw HFO counts per channel. This made our data more robust, but reduced statistical power and may have contributed to our negative result. We also used a detection threshold of 10 standard deviations rather than the three used by Brunos et al. (42), which led to a distribution of HFOs per electrode that resembled a normal rather than the expected heavy tail. While this allowed us to lateralize Subject 1 from pre-CC data, we lacked power to do so with the post-CC intervals. The threshold of three standard deviations did lateralize Subject 1 from post-CC data, but also led to incorrect lateralization of Subject 2 from pre-CC data, validating our decision to use the stricter criterion. Regardless, we strongly suggest that future studies of HFO techniques specify their parameters *a priori*. If the values for HFO detection are not established and fixed, it will remain impossible to translate techniques relying on the automatic detection of HFOs to any patient population; this is a major and pressing limitation of most studies involving HFO analysis. With this understanding, however, the inter-rater reliability and generalizability of even manual detection of HFOs has been questioned, (51, 52) [but, also, (53)] so this is not a concern limited to algorithmic methods, but may represent a constraint related to problem definition. Furthermore, while we note that the limitation of a 512 Hz sampling rate may have been insufficient to accurately measure higher-frequency phenomena, the 200 Hz 3-dB cutoff on our anti-aliasing filter is still considered in the middle of the “ripple” band (54). The observation that HFO rates may, at the group level, correlate to epileptogenicity, but that they are insufficiently specific, especially in the “ripple” frequency band, to allow for clear delimitation of the epileptogenic zone (23) or to predict an analog to the SOZ at a level superior to IEDs (24) in individual patients, is consistent with more-recent studies.

Unilateral IED frequency lateralized the more-pathologic hemisphere in all three subjects. According to a study by Lee et al., IED count in a 2-h baseline scalp EEG of patients with medial temporal lobe epilepsy predicted the pathologic hemisphere when >70% of IEDs were on one side (18). While all subjects had significantly more IEDs in their more-pathologic hemispheres, only Subject 2 had a value of Bernoulli parameter >0.70. Nevertheless, this result adds to the evidence supporting further exploration into the use of quantitative IED counting.

### Ictal Data

Weiss and coworkers used an arbitrary threshold based on a moving average of PLHG to define recruitment into a seizure. As illustrated in Figure 3A, varying the threshold changed the recruitment order considerably, including suggesting a bias toward one hemisphere or the other at various values. To avoid biasing our results, we chose to include information from a range of threshold values, from two to five standard deviations above the mean.

As expected, all subjects and metrics showed significant lateralization to the more-pathologic hemisphere post-CC, corroborating the epileptologists' reports. We were interested in whether any of the metrics predicted post-CC ictal lateralization using only pre-CC ictal data. In Subject 3, who had the best lateralization on clinician-interpreted ictal iEEG, all metrics lateralized to the more-pathologic side and there were no significant differences in the strengths of their predictions. Subject 1 showed LFLL implicating the less-pathologic hemisphere pre-CC (these seizures “tricked” the LFLL metric). Neither the epileptologist nor the high-gamma metrics were deceived—both classified the seizures as non-lateralizing. High gamma measures may contain orthogonal information to lower frequencies, making them useful adjuncts.

The PLHG measure, as implemented by Weiss et al. (55) suffers from a few technical shortcomings when applied to real-world data. Non-canonical phase-amplitude coupling (56) that results from sharp transients can manifest in PLHG similarly to epileptiform patterns, but is devoid of the oscillatory behavior that motivates the metric. The Hilbert-Huang transform is one method that future investigators may consider to overcome this issue (57), however the Gibbs phenomenon will continue to degrade data whenever it is recorded with standard anti-aliasing filters present in EEG amplifiers.

Consistent with our negative findings, Bandarabadi et al. (58), found no significant difference between the fraction of resected electrodes with supra-threshold PLHG in subjects with good (Engel I and II) and poor (Engel IV) outcomes following epilepsy surgery in a recent study, however in contrast to what would be suggested by these results, we did not see improvement in metric performance by comparing channels recruited in the first 30 s, (or expanding from the first 8 channels to the first 12) since our patients had rapidly-spreading seizures with broad multichannel recruitment. Improving on the method of calculating PLHG employed by Weiss et al. [a modification of the “phase-locking value” (27, 47)] may yield a more informative biomarker—modulation index (59), mean vector length (60), methods involving generalized linear models (48, 61) and many others, that are reviewed elsewhere (46, 62), are potential alternatives. Potential insensitivity of PLHG to early ictal changes might be related to an underlying insensitivity of the phase-locking value (46, 62), possibly because of its reliance on the phase of the analytic signal of the amplitude of another analytic signal.

### Limitations

A number of limitations were inherent to this work:

- Though the absence of clear and robust findings for high-frequency metrics from a diverse range of subjects might be informative about the real-world applicability of these findings, our *N* of 3 was too small to draw any definitive conclusions about the broader surgical epilepsy patient population. Comparing differences in the performance of PLHG, HGA, and LFLL requires combining results across subjects, and therefore larger sample sizes.
- The sampling rate of our amplifier precluded analysis of the high range of the ripple band.
- Non-canonical phase-amplitude coupling posed a challenge to the PLHG algorithm.
- Though we had widespread sampling of cortical areas across our subjects, by nature of the sEEG method we were unable to sample every brain area with an *N* of 3.
- Our data revealed the need for better/more uniform ways to define the threshold in HFO detection algorithms in order to make conclusions that are valid across studies and subjects.

### Future Directions and Clinical Implications

Our work revealed a need for prospective studies on HFO counting; retrospective studies should specifically avoid *post-hoc* HFO detector parameter selection.

Future investigators may consider that examination of HFOs co-occurring with IEDs may improve performance (63). Furthermore, research on quantitative IED counting is warranted, including with scalp recordings. Since the algorithm by Janca et al. (44), is designed and validated for intracranial data, algorithms suitable for scalp EEG will need to be tested.

## Conclusions

We tested a range of intracranial EEG biomarkers of epileptogenicity in corpus callosotomy patients, who have both ambiguous pre-CC data and ground-truth post-CC data, a novel approach for this set of biomarkers. We showed that IED counting was effective in lateralizing the more-pathologic hemisphere in 3/3 subjects with rapidly bisynchronous seizures, despite sometimes small interhemispheric differences. Replication in samples with *N*>3 would be necessary to draw broader conclusions. Interictal HFO counting correctly lateralized the more-pathologic hemisphere in one subject, but the algorithm was inconsistent and highly sensitive to changes in its parameters. PLHG and HGA were not shown to be more effective than low-frequency controls, however, they may contain information that lower-frequencies do not.

## Data Availability Statement

The raw data supporting the conclusions of this article will be made available by the authors, without undue reservation.

## Ethics Statement

The studies involving human participants were reviewed and approved by Institutional Review Board of the Feinstein Institutes for Medical Research, Northwell Health. The patients/participants provided their written informed consent to participate in this study.

## Author Contributions

SK designed and performed the analysis and wrote the manuscript. SH collected and reviewed the data and edited the manuscript. AM collected the data, planned the analysis, and edited the manuscript. All authors have approved the final manuscript.

## Funding

This work was supported by the National Institutes of Health [grant numbers NIH/NINDS NS098976-01, NIMH MH114166-01]; the National Science Foundation and the U.S.-Israel Binational Science Foundation [NSF-BSF-2017015]. None of the funding sources played a role in the design of the study; collection, analysis or interpretation of data; writing of the report; or decision to submit for publication.

## Conflict of Interest

AM was employed by P.M.T. Corporation and Medtronic. The remaining authors declare that the research was conducted in the absence of any commercial or financial relationships that could be construed as a potential conflict of interest.

## Publisher's Note

All claims expressed in this article are solely those of the authors and do not necessarily represent those of their affiliated organizations, or those of the publisher, the editors and the reviewers. Any product that may be evaluated in this article, or claim that may be made by its manufacturer, is not guaranteed or endorsed by the publisher.
